# Supplementary material for: Design of stable and self-regulated microbial consortia for chemical synthesis
Source: Nat Commun. 2022 Mar 23;13:1554. doi: 10.1038/s41467-022-29215-6 (PMC8943006; doi:10.1038/s41467-022-29215-6)
Supplement: Supplementary file 1 — Supplementary Information [file 41467_2022_29215_MOESM1_ESM.pdf]

Design of stable and self-regulated microbial consortia for chemical synthesis

Li *et. al*

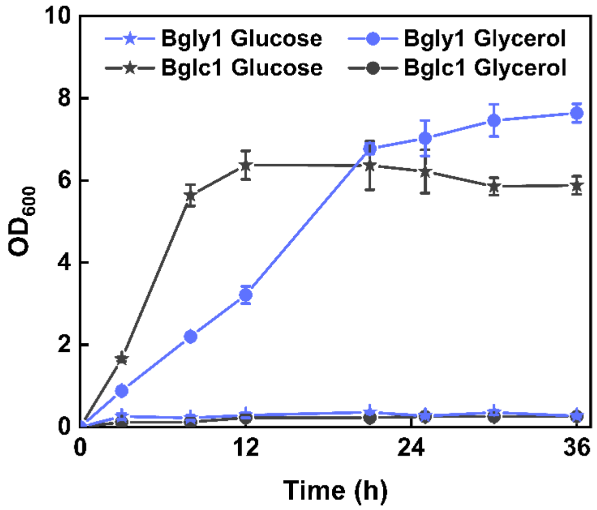

Supplementary Fig. 1. Growth of glycerol-utilizing strain Bgly1 and glucose-utilizing strain Bglc1 in glucose and glycerol media, respectively. Data shown are mean ± s.d. (n = 3 independent experiments). Source data are provided as a Source Data file.

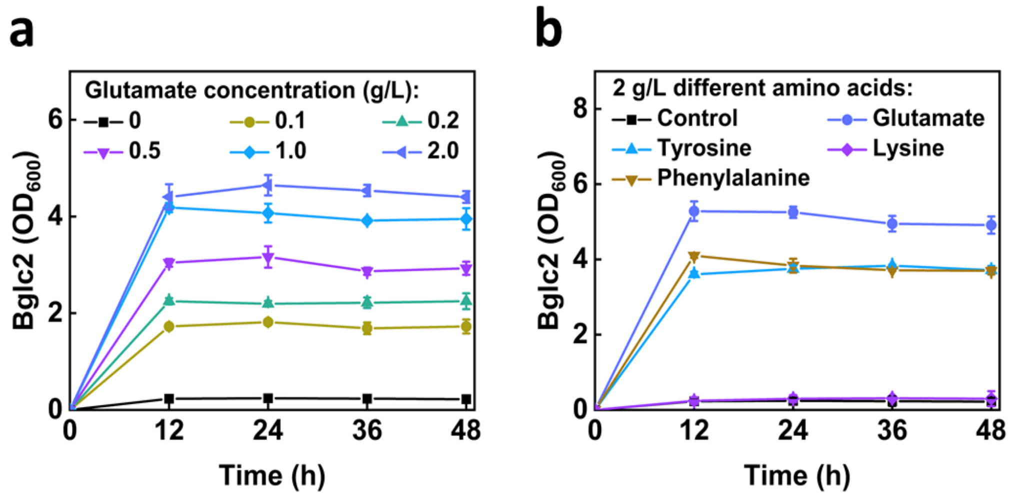

Supplementary Fig. 2. Growth of Bglc2 in culture media supplemented with different amino acids. a Growth with glutamate at different concentrations, **b** Growth with 2 g/L of each amino acid. Data shown are mean ± s.d. (n = 3 independent experiments). Source data are provided as a Source Data file.

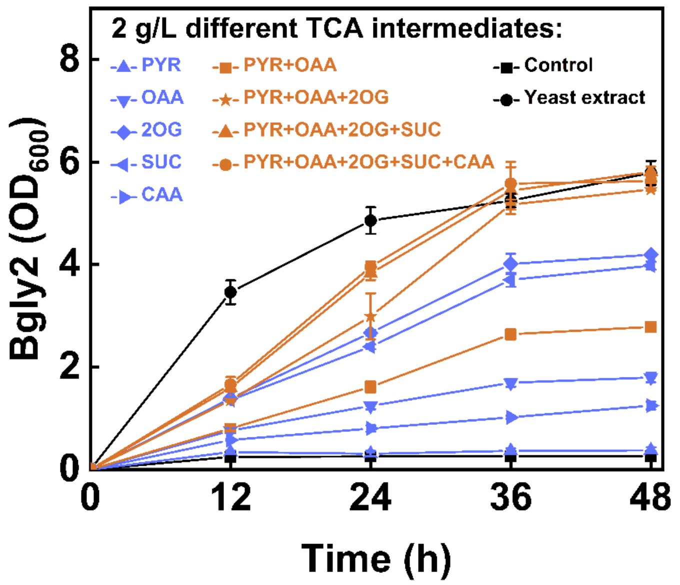

Supplementary Fig. 3. Growth curves of Bgly2 in culture media supplemented with different combinations of carboxylic acids at 2 g/L each. Data shown are mean ± s.d. (n = 3 independent experiments). Source data are provided as a Source Data file.

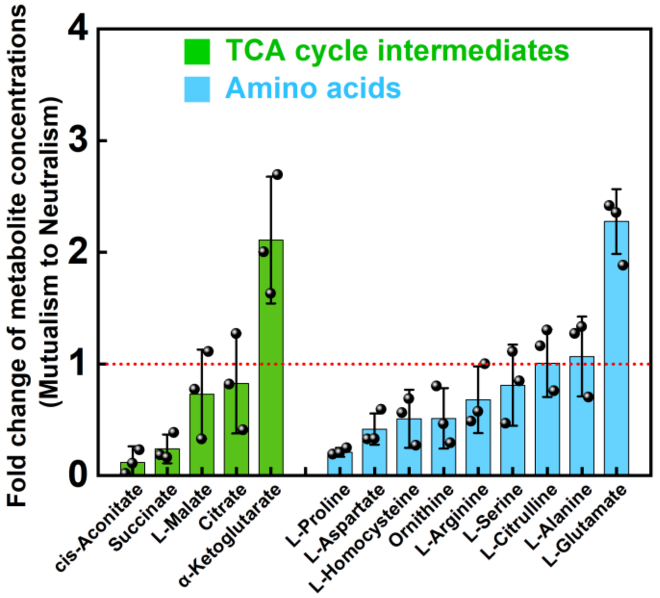

Supplementary Fig. 4. Metabolomic analysis of the supernatants of the neutral and the mutualistic cocultures. Samples were taken at 36h. Data shown are mean ± SD (n=3 independent experiments). Source data are provided as a Source Data file.

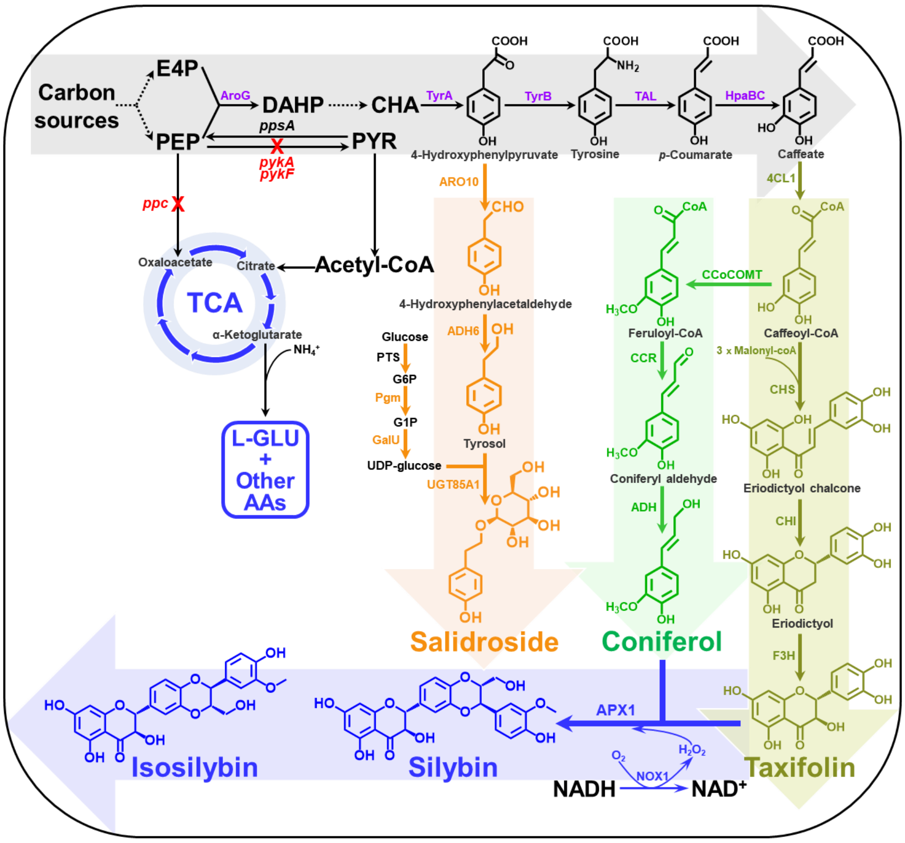

Supplementary Fig. 5. Biosynthetic pathways of salidroside, coniferol and silybin/isosilybin. Genes: *ppc* encodes phosphoenolpyruvate carboxylase; *pykA/F* encodes pyruvate kinase; *ppsA* encodes phosphoenolpyruvate synthetase; Enzymes: AroG, 3-deoxy-7-phosphoheptulonate synthase; TyrA, prephenate dehydrogenase; TyrB, aromatic amino acid transaminase; TAL, tyrosine ammonia lyase; HpaBC, 4-hydroxyphenylacetic acid 3-hydroxylase; ARO10, ketoacid decarboxylase; ADH6, alcohol dehydrogenase; UGT85A1, glycosyltransferase; Pgm, phosphoglucomutase; GalU, UDP-glucose pyrophosphorylase; 4CL1, 4-coumarate-CoA ligase; CCoAOMT, caffeoyl-CoA O-methyltransferase; CCR, cinnamoyl-CoA reductase; ADH, alcohol dehydrogenase; CHS, chalcone synthase; CHI, chalcone isomerase; F3H, flavanone 3-hydroxylase; APX1, ascorbate peroxidase 1; NOX1, NADH oxidase. Metabolites: PEP, phosphoenolpyruvate; E4P, erythrose 4-phosphate; DAHP, 3-deoxy-7-phosphoheptulonate; CHA, chorismate; PYR, pyruvate; L-GLU, L-glutamate; G6P, glucose-6-phosphate; G1P, glucose-1-phosphate; UDP-glucose, uridine diphosphate glucose.

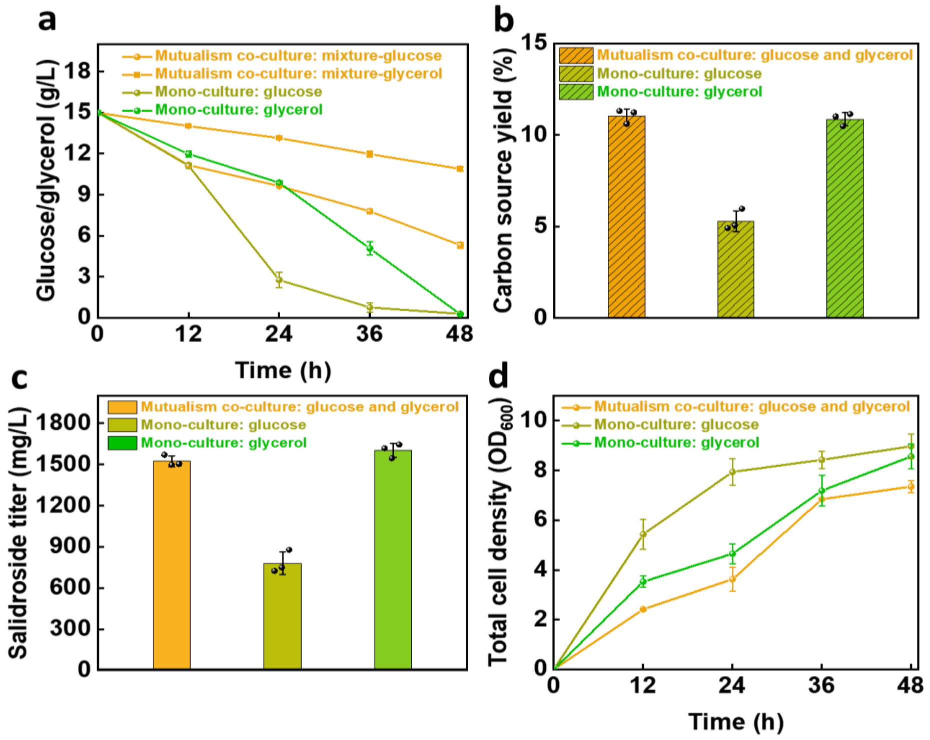

Supplementary Fig. 6. Comparison of salidroside production in the monoculture and the coculture. a Curves of carbon sources consumption, **b** Salidroside yields, **c** Salidroside titers, **d** Curves of cell growth. Strain BW-Sal was used in the monoculture while strains Bgly2-Tyr/Bglc2-Sal were used in the coculture. The IIR of the coculture is 1:1. Data shown are mean ± SD (n=3 independent experiments). Source data are provided as a Source Data file.

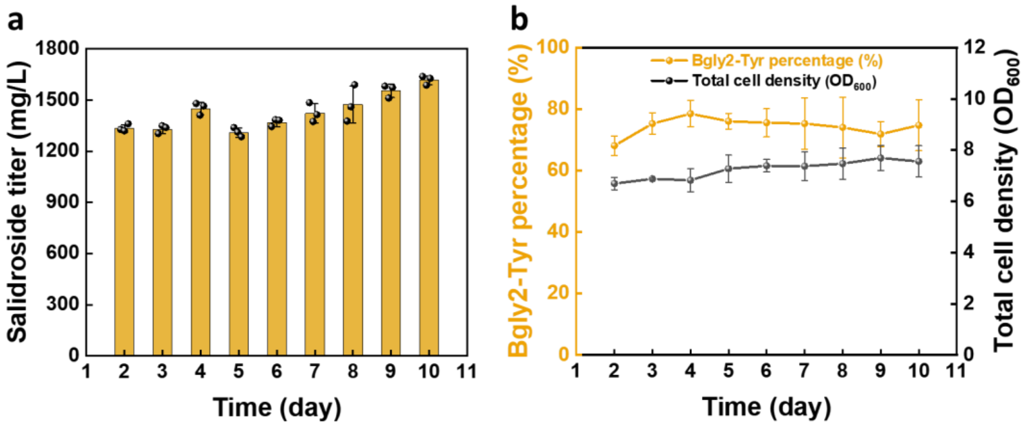

Supplementary Fig. 7. Continuous passage cultivation of the mutualistic coculture. a Salidroside titers, and **b** Population composition of each subculture. Data shown are mean ± SD (n=3 independent experiments). Source data are provided as a Source Data file.

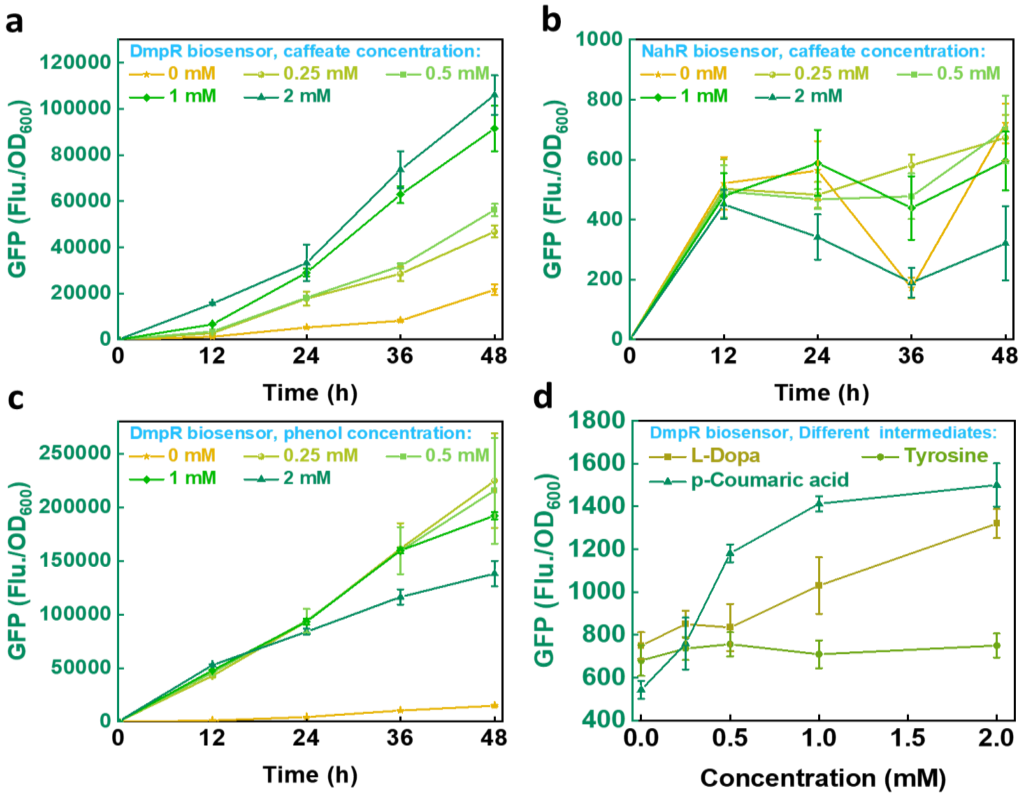

Supplementary Fig. 8. Responsiveness of DmpR and NahR biosensors to different phenolic compounds. a DmpR to caffeate, **b** NahR to caffeate, **c** DmpR to phenol and **d** DmpR to L-dopa, tyrosine and *p*-coumaric acid**.** Data shown are mean ± s.d. (n = 3 independent experiments). Source data are provided as a Source Data file.

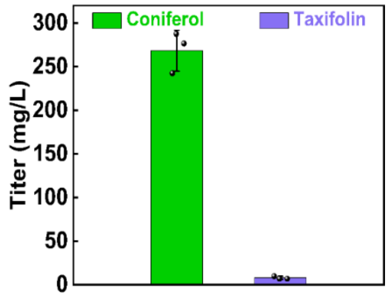

Supplementary Fig. 9. Production of coniferol and taxifolin from caffeate. Strains BW21513 (pZE-CA4C) and BW21513 (pZE-CCF4) were used. Caffeate (500 mg/L) was fed to the cell cultures, and samples were taken at 48 h. The Data shown are mean ± s.d. (n = 3 independent experiments). Source data are provided as a Source Data file.

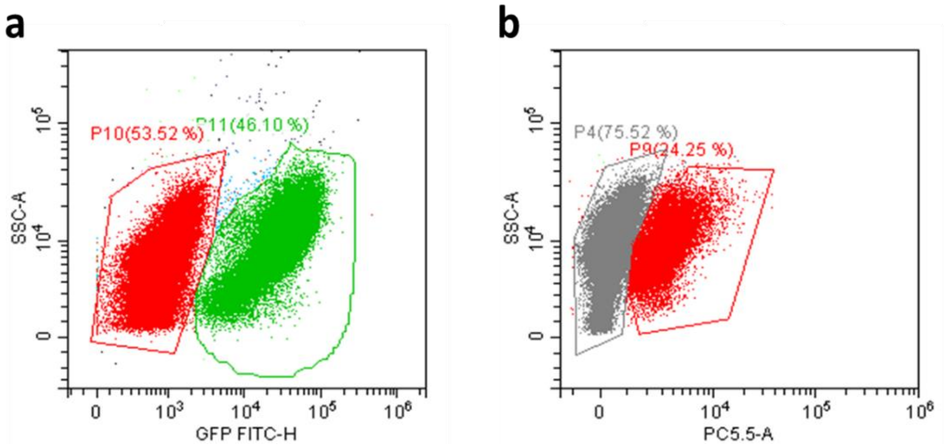

Supplementary Fig. 10. Two examples of the flow cytometry results. a Analysis of the population composition with green and red fluorescence. **b** Analysis of the population composition with/without red fluorescence. SSC-A, Side Scatter-Area; FITC-H, Fluoresce in Isothiocyanate-Height; PC5.5-A, Phycoerythrin Cyanin 5-Area.

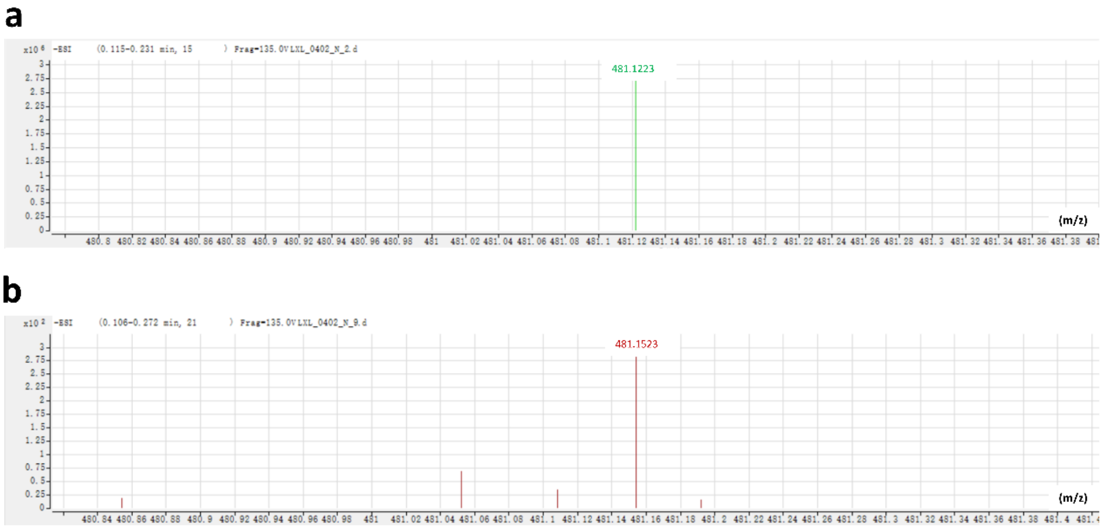

Supplementary Fig. 11. ESI-MS results of silybin/isosilybin. a The standard, **b** The sample. Source data are provided as a Source Data file.

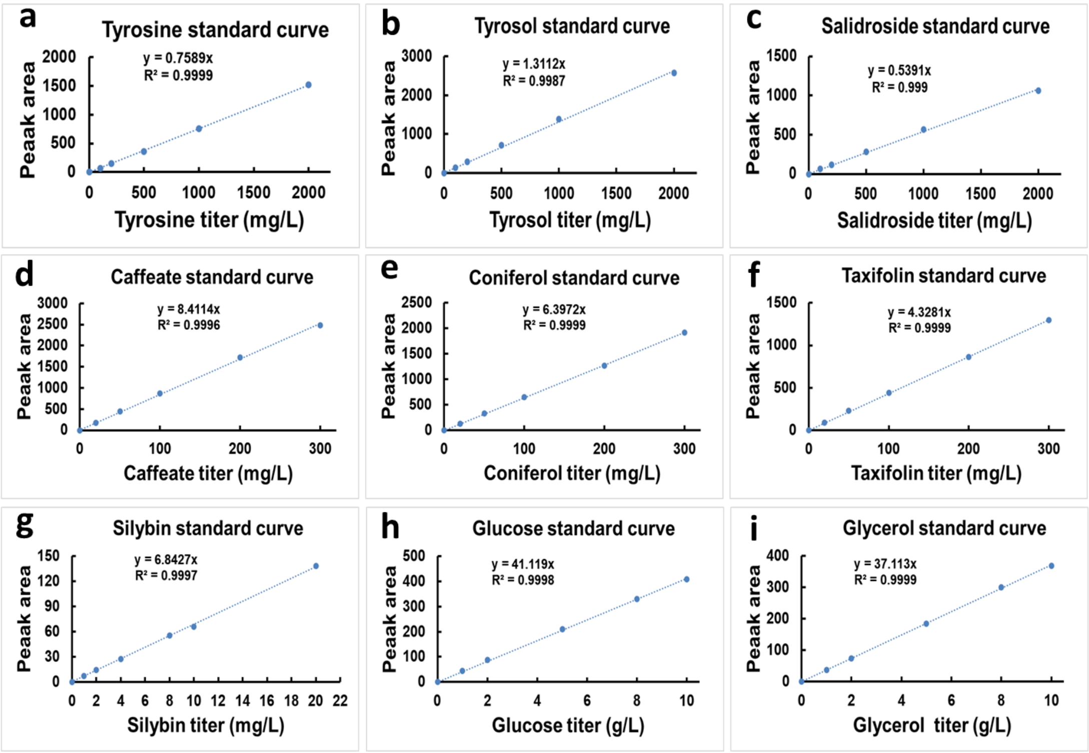

Supplementary Fig. 12. Standard curves of the related compounds in HPLC. a Tyrosine, **b** Tyrosol, **c** Salidroside, **d** Caffeate, **e** Coniferol, **f** Taxifolin, **g** Silybin/isosilybin, **h** Glucose and **i** Glycerol. Source data are provided as a Source Data file.

Supplementary Table 1. Strains used in this study.

Strains	Description	Source
BW25113	*rrnBT*14 Δ*lacZWJ*16 *hsdR*514 Δ*araBADAH*33 Δ*rhaBADLD*78	Coli genome stock center
BW25113Δ*pykAF*	BW25113Δ*pykA*Δ*pykF*	1
Bgly1	BW25113Δ*pykA*Δ*pykF*Δ*ptsG*Δ*glk*Δ*manXYZ*	This study
Bglc1	BW25113Δ*glpk*	This study
Bgly2	BW25113Δ*pykA*Δ*pykF*Δ*ptsG*Δ*glk*Δ*manXYZ*Δ*ppc*	This study
Bglc2	BW25113Δ*glpk*Δ*gdhA*Δ*gltBD*	This study
Bgly1-Tyr	Bgly1, pZE12-luc and pCS-TPTA and pSA-mcherry	This study
Bglc1-Sal	BGlc1, pZE-ugt85A1 and pCS-pg and pSA-AA	This study
Bgly2-Tyr	Bgly2, pZE12-luc and pCS-TPTA and pSA-mcherry	This study
Bglc2-Sal	Bglc2, pZE-ugt85A1 and pCS-pg and pSA-AA	This study
BW-Sal	BW25113Δ*pykAF*, pZE-ugt85A1 and pCS-TPTA-pg and pSA-AA	This study
Bgly2-Caf	Bgly2, pZE-TAL and pCS-TH and pSA-mcherry	This study
Bglc2-Con	Bglc2, pZE-CA4C and pCS27 and pSA-eGFP	This study
Bglc2-ConDmpR	Bglc2, pZE-CA4C and pCS27 and pSA-P_{dmp}-GS-P_{J23101}-dmpR	This study
Bglc2-Sil	Bglc2, pZE-CA and pCS-CF4C-P_{J23101}-CA and pSA-P_{dmp}-GS-P_{J23101}-DN	This study
Bglc2-Con(b)	Bglc2, pZE12-luc and pCS-4C-P_{J23101}-CA and pSA-eGFP	This study
Bglc2-Sil(b)	Bglc2, pZE-CA and pCS-CF4 and pSA-P_{dmp}-GS-P_{J23101}-DN	This study

Supplementary Table 2. Plasmids used in this study.

Plasmids	Description	Source
pZE12-luc	$P_L$lacO1, *colE ori, luc, Amp*^{R}	1
pCS27	$P_L$lacO1, *P15A ori, Kan*^{R}	1
pSA74	$P_L$lacO1, *pSC101 ori, Cl*^{R}	1
pCS-TPTA	pCS27, *tyrA, ppsA, tktA* and *aroG* from *E. coli*	1
pSA-mcherry	pSA74, *mcherry*	This study
pZE-ugt85A1	pZE12-luc, *ugt85A1* from *Arabidopsis thaliana*	This study
pCS-pg	pCS27, *pgm* and *galU* from *E. coli*	This study
pSA-AA	pSA74, *aro10* and *adh6* from *Saccharomyces cerevisiae*	This study
pZE-TAL	pZE12-luc, *tal* from *Rhodobacter glutinis*	2
pCS-TH	pCS27, *tyrA, ppsA, tktA, aroG* and *hpaBC* from *E. coli*	1
pZE-CCF4	pZE12-luc, *chs* from *Petunia x hybrida, chi* from *Medicago sativa, f3h* from *Camellia sinensis* and *4cl1* from *A. thaliana*	This study
pZE-CA4C	pZE12-luc, *ccr* from *Leucaena leucocephala, adh* from *S.cerevisiae, 4cl1, ccoaomt* from *A. thaliana*	2
pSA-eGFP	pSA74, *egfp*	This study
pSA- P_{J23101}-dmpR	pSA74, P_{J23101} promoter, *dmpR* from *Pseudomonas CF600*	This study
pSA- P_{J23101}-nahR	pSA74, P_{J23101} promoter, *nahR* from *Pseudomonas putida*	This study
pZE-P_{dmp}-eGFP	pZE12-luc, *egfp* with P_{dmp} promoter	This study
pZE-P_{nah}-eGFP	pZE12-luc, *egfp* with P_{nah} promoter	This study
pSA-P_{dmp}-GS-P_{J23101}-dmpR	pSA74, *gdhAssrA* from *E. coli* with P_{dmp} promoter and *dmpR* with P_{J23101} promoter	This study
pSA-P_{dmp}-GS-P_{J23101}-DN	pSA74, *gdhA*ssrA with P_{dmp} promoter, *dmpR* and nox1 (from *Lactococcus lactis*) with P_{J23101} promoter	This study
pZE-APX1	pZE12-luc, *apx*1 from *Silybum marianum*	This study
pCS-P_{lac}-CF4C-P_{J23101}-CA	pCS27， *chi, f3h, 4cl*1 and *ccoaomt* with P_{lac} promoter, *ccr* and *adh* with P_{J23101} promoter	This study
pZE-CHS	pZE12-luc, *chs*	This study
pCS-CF4	pCS27， *chi, f3h* and *4cl1*	This study
pZE-CA	pZE12-luc, *chs and apx1*	This study
pCS-4C-P_{J23101}-CA	pCS27， *4cl1* and *ccoaomt* with P_{lac} promoter, *ccr* and *adh* with P_{J23101} promoter	This study
pSA-P_{J23101}-NOX1	pSA74, *nox1* with P_{J23101} promoter	This study

Supplementary references

1. Li, X. et al. Establishing an artificial pathway for efficient biosynthesis of hydroxytyrosol. *ACS Synth Biol* **7**, 647-654 (2018).

2. Chen, Z., Sun, X., Li, Y., Yan, Y. & Yuan, Q. Metabolic engineering of *Escherichia coli* for microbial synthesis of monolignols. *Metab Eng* **39**, 102-109 (2017).
