# Supplementary material for: Design of stable and self-regulated microbial consortia for chemical synthesis
Source: Nat Commun. 2022 Mar 23;13:1554. doi: 10.1038/s41467-022-29215-6 (PMC8943006; doi:10.1038/s41467-022-29215-6)
Supplement: Supplementary file 3 — Description of Additional Supplementary Files [file 41467_2022_29215_MOESM3_ESM.pdf]

Description of Additional Supplementary Files

File Name: Supplementary Data 1
Description: Oligonucleotides used in this study
