# Supplementary material for: Design of stable and self-regulated microbial consortia for chemical synthesis
Source: Nat Commun. 2022 Mar 23;13:1554. doi: 10.1038/s41467-022-29215-6 (PMC8943006; doi:10.1038/s41467-022-29215-6)
Supplement: Supplementary file 5 — Reporting Summary [file 41467_2022_29215_MOESM5_ESM.pdf]

nature portfolio

Corresponding author(s): Xinxiao Sun

Last updated by author(s): Feb 25, 2022

Reporting Summary

Nature Portfolio wishes to improve the reproducibility of the work that we publish. This form provides structure for consistency and transparency in reporting. For further information on Nature Portfolio policies, see our Editorial Policies and the Editorial Policy Checklist.

Statistics

For all statistical analyses, confirm that the following items are present in the figure legend, table legend, main text, or Methods section.

n/a	Confirmed	
☐	☒	The exact sample size (*n*) for each experimental group/condition, given as a discrete number and unit of measurement
☐	☒	A statement on whether measurements were taken from distinct samples or whether the same sample was measured repeatedly
☒	☐	The statistical test(s) used AND whether they are one- or two-sided<br>*Only common tests should be described solely by name; describe more complex techniques in the Methods section.*
☒	☐	A description of all covariates tested
☒	☐	A description of any assumptions or corrections, such as tests of normality and adjustment for multiple comparisons
☐	☒	A full description of the statistical parameters including central tendency (e.g. means) or other basic estimates (e.g. regression coefficient) AND variation (e.g. standard deviation) or associated estimates of uncertainty (e.g. confidence intervals)
☒	☐	For null hypothesis testing, the test statistic (e.g. *F*, *t*, *r*) with confidence intervals, effect sizes, degrees of freedom and *P* value noted<br>*Give P values as exact values whenever suitable.*
☒	☐	For Bayesian analysis, information on the choice of priors and Markov chain Monte Carlo settings
☒	☐	For hierarchical and complex designs, identification of the appropriate level for tests and full reporting of outcomes
☒	☐	Estimates of effect sizes (e.g. Cohen's *d*, Pearson's *r*), indicating how they were calculated

Our web collection on statistics for biologists contains articles on many of the points above.

Software and code

Policy information about availability of computer code

Data collection	CytExpert 2.0 was used to collect the data of the flow cytometry experiment.
Data analysis	OriginPro 9.0 was used for data analysis.

For manuscripts utilizing custom algorithms or software that are central to the research but not yet described in published literature, software must be made available to editors and reviewers. We strongly encourage code deposition in a community repository (e.g. GitHub). See the Nature Portfolio guidelines for submitting code & software for further information.

Data

Policy information about availability of data

All manuscripts must include a data availability statement. This statement should provide the following information, where applicable:

- Accession codes, unique identifiers, or web links for publicly available datasets
- A description of any restrictions on data availability
- For clinical datasets or third party data, please ensure that the statement adheres to our policy

The data generated in this study are provided in the Supplementary Information/Source Data file.

Field-specific reporting

Life sciences study design

All studies must disclose on these points even when the disclosure is negative.

Sample size	To monitor the stability and calculate the standard deviation, the fermentation experiments were performed in triplicate by picking three random colonies of each strain.
Data exclusions	A very small portion of data (<5%) were excluded because they significantly deviated from the corresponding averages.
Replication	Each experiment is performed at least twice, confirming the reproducibility of the experiments.
Randomization	Colonies are picked randomly from the agar plates.
Blinding	Blinding is generally not necessary for the microbial fermentation experiments.

Reporting for specific materials, systems and methods

We require information from authors about some types of materials, experimental systems and methods used in many studies. Here, indicate whether each material, system or method listed is relevant to your study. If you are not sure if a list item applies to your research, read the appropriate section before selecting a response.

Materials & experimental systems

n/a	Involved in the study
☒	☐ Antibodies
☒	☐ Eukaryotic cell lines
☒	☐ Palaeontology and archaeology
☒	☐ Animals and other organisms
☒	☐ Human research participants
☒	☐ Clinical data
☒	☐ Dual use research of concern

Methods

n/a	Involved in the study
☒	☐ ChIP-seq
☐	☒ Flow cytometry
☒	☐ MRI-based neuroimaging

Flow Cytometry

Plots

Confirm that:

☒ The axis labels state the marker and fluorochrome used (e.g. CD4-FITC).

☒ The axis scales are clearly visible. Include numbers along axes only for bottom left plot of group (a 'group' is an analysis of identical markers).

☒ All plots are contour plots with outliers or pseudocolor plots.

☒ A numerical value for number of cells or percentage (with statistics) is provided.

Methodology

Sample preparation	Samples (E.coli cell cultures) was diluted 1000 times and placed in a 2 ml collection tube after thorough mixing.
Instrument	Beckman CytoFlexS was used for data collection.
Software	CytExpert 2.0 was used to collect data, and originPro 9.0 was used to analyze data.
Cell population abundance	Flow cytometry was used to calculate the percentage of each strain in the cocultures.
Gating strategy	The total number of assays was set to 100000, the sample flow rate was medium (30 µL/min), and the cell granularity threshold was >800. Isothiocyanate (FITC, 525/40) and Phycoerythrin Cyanin 5 (PE-Cy5, 690/50) filters were used to screen for cells with green and red fluorescence, respectively.

☒ Tick this box to confirm that a figure exemplifying the gating strategy is provided in the Supplementary Information.
